# Quantitative Imaging With DNA-PAINT for Applications in Synaptic Neuroscience

**DOI:** 10.3389/fnsyn.2021.798267

**Published:** 2022-02-07

**Authors:** Eduard M. Unterauer, Ralf Jungmann

**Affiliations:** ^1^Max Planck Institute of Biochemistry, Martinsried, Germany; ^2^Faculty of Physics and Center for Nanoscience, Ludwig Maximilian University, Munich, Germany

**Keywords:** DNA-PAINT, DNA nanotechnology, neuronal target, fluorescence microscopy, super-resolution microscopy

## Abstract

Super-resolution (SR) microscopy techniques have been advancing the understanding of neuronal protein networks and interactions. Unraveling the arrangement of proteins with molecular resolution provided novel insights into neuron cytoskeleton structure and actin polymerization dynamics in synaptic spines. Recent improvements in quantitative SR imaging have been applied to synaptic protein clusters and with improved multiplexing technology, the interplay of multiple protein partners in synaptic active zones has been elucidated. While all SR techniques come with benefits and drawbacks, true molecular quantification is a major challenge with the most complex requirements for labeling reagents and careful experimental design. In this perspective, we provide an overview of quantitative SR multiplexing and discuss in greater detail the quantification and multiplexing capabilities of the SR technique DNA-PAINT. Using predictable binding kinetics of short oligonucleotides, DNA-PAINT provides two unique approaches to address multiplexed molecular quantification: qPAINT and Exchange-PAINT. With precise and accurate quantification and spectrally unlimited multiplexing, DNA-PAINT offers an attractive route to unravel complex protein interaction networks in neurons. Finally, while the SR community has been pushing technological advances from an imaging technique perspective, the development of universally available, small, efficient, and quantitative labels remains a major challenge in the field.

## Introduction

In recent years, SR microscopy has been a rising technique to investigate complex biological systems and molecular mechanisms. With the availability of site-specific labeling and nanometer-scale resolutions, SR microscopy enables mapping of cellular components and single-cell heterogeneity with near molecular resolution. The investigation of neuronal tissue sections presents a unique challenge for imaging techniques. Signal transduction in this complex cellular network occurs in synaptic junctions, in which large networks of protein species are orchestrated in the space of just a few hundred nanometers. Signaling at synapses is mediated by pools of synaptic vesicles just 50 nm in size. However, they contain hundreds of surface proteins (Takamori et al., 2006). Thus, these objects exhibit high molecular densities due to a large amount of protein copy numbers clustered in a relatively small area. To reliably identify key interactor proteins and place them into the context of larger structures, SR microscopy needs to facilitate accurate mapping of these protein networks and provide reliable molecule numbers for quantification. Both illumination-based SR techniques such as Structured illumination microscopy (SIM) (Gustafsson, 2005) or Stimulated Emission depletion microscopy (STED) (Hell and Wichmann, 1994) and localization-based SR techniques like Photoactivated-localization microscopy (PALM) (Betzig et al., 2006) or stochastic optical reconstruction microscopy (STORM) (Rust et al., 2006) have been used to study neuronal targets. The most prominent examples being the investigation of dendritic spine dynamics in living mice (Berning et al., 2012) by STED and the mapping of periodic Actin-Spectrin filaments in fixed mouse hippocampal neurons by STORM (Xu et al., 2012). While enabling unprecedented spatial resolution and quantification capabilities, SR techniques have advantages and disadvantages, with the obligation of the researcher to decide, which technique fulfills the right requirements for a given biological question under investigation. Here, we will discuss the possibilities and caveats of the localization-based SR technique DNA Points Accumulation in Nanoscale Topography (DNA-PAINT) (Jungmann et al., 2010) for quantitative and multiplexed investigation of neuronal targets.

## Super-Resolution Microscopy With DNA-Paint

All localization-based SR techniques aim to separate the detection of individual fluorescent molecules in space and time. The achievable localization precision in these approaches is ultimately limited by the total amount of photons and exhibits $1/\sqrt{n_{p\,h\,o\,t\,o\,n\,s}}$ scaling, with *n*_*photons*_ being the number of detected photons that can be detected from a single blinking or binding (Thompson et al., 2002). In contrast to PALM or STORM, where fluorescent proteins or organic dyes with photoswitching capabilities are employed, PAINT uses a different approach (Sharonov and Hochstrasser, 2006). Here, freely diffusing dyes or dye-labeled ligands that transiently interact with their targets are used to achieve the necessary molecular “blinking.” In the case of DNA-PAINT, these ligands are small 6–10 nucleotides (nt) long single-stranded (ss) DNA strands, called imager strands, which bind to their complementary ssDNA strands called docking strands on a target. In comparison to other SR techniques, DNA-PAINTs advantages are the high sub-5-nm spatial resolution and—due to the technically infinite pool of imager sequences—negligible photobleaching (Dai et al., 2016). Additionally, as “blinking” is decoupled from the photophysical dye properties, DNA-barcoded targets allow for quantification and multiplexing with qPAINT (Jungmann et al., 2016) and Exchange-PAINT (Jungmann et al., 2014). However, the most severe drawbacks of DNA-PAINT have traditionally been long image acquisition times and the need for selective plane illumination due to the non-fluorogenic nature of the imager strands in solution.

## Quantitative DNA-PAINT (qPAINT) for Synaptic Targets

Understanding the molecular mechanisms underlying complex neuronal structures and signaling pathways requires investigations on the level of single molecules. Only when single emitters can be resolved, a complete picture of the spatial arrangement and quantitative numbers can be obtained for each relevant protein target. Out of all possible targets in neurons, the synapse presents itself as a particularly interesting entity for quantitative SR studies. The delicate machinery of signal transduction is orchestrated in a space of only a few hundreds of nanometers and varies among different types of synapses, such as inhibitory or excitatory. Furthermore, the spatial arrangement and composition of proteins in the synapses as well as its structure and plasticity is changing in important neurodegenerative and autoimmune diseases (Ladepeche et al., 2018; Jackson et al., 2019).

**FIGURE 1:**
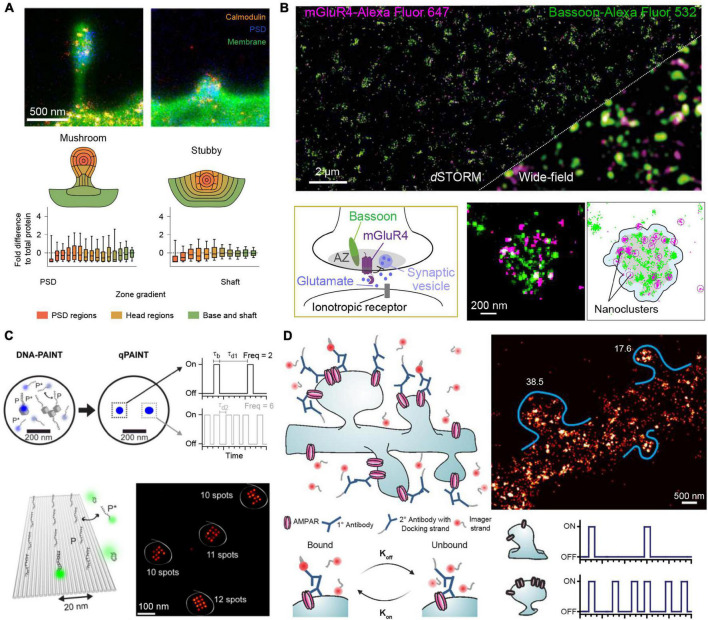
Quantitative SR imaging of neuronal targets. **(A)** STED nanoscopy for quantification of Calmodulin protein copy numbers in dendritic spines. The top shows examples for mushroom-like and stubby dendritic spines visualized by the membrane stain DiO in green, the Postsynaptic density by Homer1 in blue and the protein of interest, Calmodulin, in orange. The bottom shows the protein enrichment separated into regions of the dendritic spines, in total *n* = 150 mushroom-like and *n* = 140 stubby spines were analyzed showing an overall similar distribution for both spine types. Adapted with permission (Helm et al., 2021). **(B)** *d*STORM microscopy for determining mGluR4 clusters at synaptic active zones. Top shows a comparison of a two-color *d*STORM image of mGluR4 (Purple) and the presynaptic active zone marker Bassoon (green) to the respective diffraction-limited image. Bottom shows a schematic of the mGluR4 located at the presynaptic active zone (AZ) and DBSCAN cluster analysis of Bassoon and mGluR4 for determining the area (gray) of the AZ and mGluR4 nanoclusters. Adapted with permission (Siddig et al., 2020). **(C)** qPAINT implementation. In DNA-PAINT, fluorescently labeled “imager” strands (P*) transiently bind from solution to complementary “docking” strands (P) attached to a target. Intensity vs. time traces show characteristic fluorescence on- and off-times (τ_*b*_ and τ_*d*_, respectively). qPAINT uses the predictable blinking kinetics to deduct absolute molecule numbers. Top shows two exemplary regions imaged by DNA-PAINT and evaluated by qPAINT. From a single emitter (single gray cube) the imager-specific kinetics, the bright time and dark time can be extracted. Afterward this extracted dark time can be used to calculate the number of single emitters in a more crowded region where molecular resolution cannot be achieved (three cubes). Bottom shows the respective results in an exemplary 12 binding site DNA origami surface, where qPAINT correctly predicts the amount of available binding sites per structure. Adapted with permission (Jungmann et al., 2016). **(D)** Quantification of AMPA receptor complexes by qPAINT on GluA2 receptors. Top left shows an illustration of DNA-PAINT labeling and imaging of dendritic spines via primary and secondary antibodies. Bottom: By analyzing the kinetic traces of subregions of the dendrites, molecular counting can be achieved by comparing the average dark time for the regions to single binding sites for calibration. Top right shows the qPAINT results for two dendritic spines. Adapted with permission (Boger et al., 2019).

Recent studies have used nanoscopy methods to characterize 110 different proteins (albite in different samples) in dendritic spines and obtained ensemble numbers for each protein species (Helm et al., 2021). Combining STED nanoscopy and electron microscopy, Helm et al. (2021) have visualized protein distributions of stubby and mushroom-like dendritic spines (Figure 1A). They were able to show that while both types contain on average similar protein numbers and topology, stubby spines express a lower number of trafficking-related proteins in correlation to the postsynaptic density mass, indicating a lower dynamic response of those spines. This corresponds well to the general hypothesis that stubby spines represent a rather immature developmental state, while mushroom-like spines compartmentalize receptors and proteins for signal transduction cascades. (Harris et al., 1992; Berry and Nedivi, 2017) Apart from using electron microscopy as a reference, more studies use antibody titration measurements for comparison and validation of the super-resolved data. Siddig et al. (2020) determined the distribution of metabotropic glutamate receptor 4 (mGluR4) in cerebellar active zones (Figure 1B) and analyzed their colocalization with Cav2.1 calcium channel and the presynaptic scaffold marker Bassoon. Using direct STORM (dSTORM) super-resolution microscopy combined with cluster analysis, Siddig et al. (2020) were able to show that all three proteins in fact do colocalize. This colocalization of mGluR4 nanodomains in the active zone with Cav2.1 channels suggests that mGluR4 might regulate neurotransmitter release by influencing Calcium influx. The mGluR4 copy numbers per active zone were determined to be about 35 and the data was validated by using a ramp of different antibody concentrations and fitting the resulting to a logistic function. While the assignment of molecule numbers with sparse protein targets is a relatively straight forward, this becomes exceedingly more complex in more crowded environments of highly expressed proteins.

Quantitative counting approaches using e.g., STORM or PALM as imaging modality suffer from potential over- or undercounting due to unpredictable and hard-to-calibrate photophysics of photoswitchable proteins and organic dyes, leading to downstream quantification artifacts. DNA-PAINT on the other hand offers a distinct way to deduct integer numbers of molecules from analyzing blinking kinetics of transient DNA hybridization, which enables precise and accurate counting, as the blinking fingerprint is largely independent from photophysical properties of dye molecules. This approach is called Qpaint (Jungmann et al., 2016) and uses the predictable second-order association kinetics of imager strands to their docking strands to obtain quantitative molecule numbers (Figure 1C). In brief, first the influx rate ξ=*k*_*on*_⋅*c*_*i*_ for imager strands to targets is calibrated with a sample containing a known number of binding sites. Here, *k*_*on*_ represents the association rate for the hybridization of imager to docking strands and *c_i* the concentration of imager strands. In a second step, the influx rate in an analysis area with unknown quantity of binding sites (and thus target molecules) is determined. As ξ scales linearly with the number of target strands, integer numbers of molecules can be determined with qPAINT. Jungmann et al. (2016) first established this approach using DNA origami nanostructures (Figure 1C). As an origami can be designed with a prescribed number of docking strands, it can serve as an exquisite ground truth for developing and benchmarking new single-molecule approaches, before applying the tried and tested technique in more complex *in situ* environments (e.g., inside a cell). Optimizing acquisition conditions yielded a high qPAINT detection accuracy and precision. As a next step, the method was applied to count Bruchpilot (Brp) proteins in drosophila neuromuscular junction synaptic active zones. The individual Brp protein clusters were too dense to spatially resolve single binding sites. However, qPAINT analysis could determine ∼142 Brp molecules per cluster, in good agreement with numbers reported from an earlier study (Ehmann et al., 2014).

In a more recent study, Boger et al. (2019) were able to determine the average copy numbers of GluA2 molecules, a subpart of the AMPA-type glutamate receptor complex (AMPAR), in mouse hippocampal dendritic spines (Figure 1D). To optimize qPAINT for their system, the authors first performed *in silico* and *in vitro* optimizations using DNA origami structures mimicking the expected distribution and numbers of GluA2 molecules. Their simulated data yielded a detection efficiency of 84%, analyzing clusters with 40 and 15 nm docking site spacing. Their subsequent qPAINT application in dendritic spines yielded an average of 23 molecules per spine, again in good agreement with earlier studies (Nair et al., 2013). With the rise of quantitative super-resolution microscopy, it is now possible to obtain precise protein number distributions and with the help of advanced cluster analysis approaches, infer downstream mechanistic information.

Compared to more incumbent single-molecule localization microscopy counting approaches, qPAINT offers a unique advantage of immunity to under- and overcounting biases, thanks to its reliance on the predictable binding kinetics of DNA molecules to their complements and the largely photobleaching free image acquisition process. Furthermore, as qPAINT (similar to DNA-PAINT) decouples the apparent blinking from the photophysical properties of dyes, it is easily multiplexable (Jungmann et al., 2016). One potential caveat, however, might arise by inaccuracies due to altered binding kinetics in dense cellular environments such as the cell nucleus, but recent calibration-free advances could alleviate this (Stein et al., 2021).

More generally, it is important to consider that although on a conceptual level (and for *in vitro* experiments using e.g., DNA origami structures for quantification) accuracy and precision of qPAINT is excellent, this unfortunately does not hold true for the case of most cellular applications. A critical performance-determining factor in cellular applications is the efficiency and specificity of the employed labeling probes used to tag target molecules of interest with a fluorophore or DNA strand. This efficiency and specificity crucially influence the final accuracy of the imaging and counting approach for e.g., visualization and quantification of proteins in cells.

The main challenges for quantitative immunolabeling are twofold: Can we assume that the location of the emitter is a truthful representation of the target position? And second, is the number of emitters a good proxy for the true number of targets?

The two main determining factors regarding those two issues are: *(1) The size of the labeling probe*. The smaller the probe, the lower the so-called “linkage” error, which in turn leads to a more accurate representation of the true target position. *(2) The labeling stoichiometry between labeling probe and target*. Ideally, one would aim for a 1:1 labeling stoichiometry of probe to target for the most accurate and precise quantification.

The most common approach for immunolabeling are species- and host-dependent pairs of primary and secondary antibodies. While well established and available for many targets, this labeling approach unfortunately results in a relatively large probe sandwich and thus linkage error (approx. 20–25 nm) rendering not ideal for super-resolution microscopy (Tang et al., 2016; Moore and Legant, 2018; Ganji et al., 2021). Furthermore, multiple secondary antibodies (carrying potentially more than one dye or DNA strand) make precise and accurate quantification difficult. While this “amplification” effect of a target signal (due to linking many primary amines or thiol groups of antibodies to dyes or DNA) is in fact advantageous for increased signal-to-background in e.g., confocal microscopy applications, it is not ideal for absolutely quantitative single-molecule studies. However, while not enabling highest counting precision, good counting accuracy can still be achieved when single, spatially separated targets are used for calibration.

To improve labeling stoichiometry and probe size while still maintaining advantages of primary antibodies (e.g., widespread availability), labeling with secondary nanobodies has recently been introduced. Secondary nanobodies are designed to carry only a single C-terminal Cysteine for dye or DNA coupling and thus allow for a much-improved precision in counting (Pleiner et al., 2015). Additionally, being only 3 nm in size, these small secondary labeling probes significantly decrease the linkage error and show improved labeling efficiency. Finally, primary antibodies can be conjugated directly with dyes or DNA molecules, however, with potentially adverse side-effects such as reduced target binding affinity when non-site-specific labeling approaches are chosen. However, if carefully optimized, direct conjugation of primary antibodies not only “saves” the step of secondary antibody or nanobody incubation, but—more importantly—it prevents species-dependent crosstalk of antibodies in multiplexing applications.

While these labeling approaches discussed above are most common to date, substantial progress has been made in the quantitative and efficient labeling of a handful of important targets, which we will discuss later.

## Implementation of Quantitative Super-Resolution Multiplexing

Most quantitative SR investigations of proteins in neurons include one or several rounds of multiplexing with diffraction-limited reference targets, most prominently PSD95 or Synapsin, for identification of dendritic spines or synapses, and MAP2 for mapping the neuronal geometry. For SR quantification of multiple protein targets, spectrally distinct dyes are usually employed. However, only few optimized dye combinations are available (Dempsey et al., 2011). While this approach is relatively straightforward, the multiplexing capacity is limited by the spectral overlap of dyes. Furthermore, since many dyes are not suited for certain super-resolution approaches (Dempsey et al., 2011), a common approach to investigate multiple targets is to use one wavelength as reference and map the target localizations relative to this reference (Agasti et al., 2017). This approach has advantages and disadvantages: Imaging one important target with super-resolution in each sample allows for optimized staining and fixation conditions tailored to this specific target. Also examining one sample at a time avoids complex experimental designs for multiplexed immunostaining or automated liquid handling at the microscope.

**FIGURE 2:**
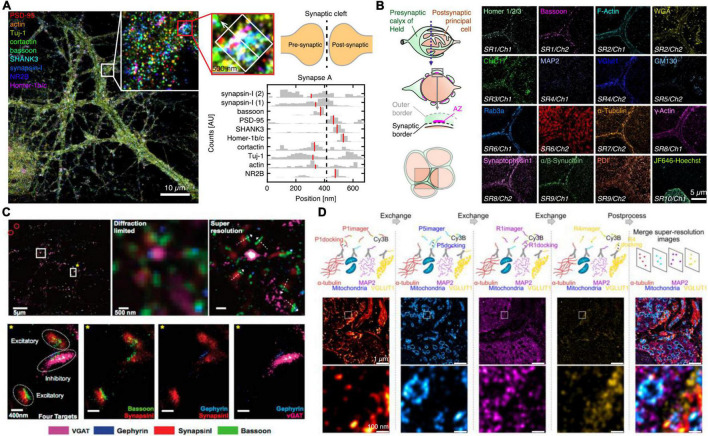
Multiplexed SR imaging of neuronal targets. **(A)** Super-resolved DNA-PRISM multiplexing of synaptic targets. Left shows an overlayed display of nine targets imaged in neuronal cultures. The targets involve five synaptic proteins, three cytoskeleton proteins and the glutamate receptor subunit NR2B. The right shows cross-sectional profiles of the highlighted individual synapse with the median of the distributions indicated in red. Adapted with permission (Guo et al., 2019). **(B)** Automated maS^3^TORM imaging of 16 targets in the giant calyx of Held synapse. The targets were subsequently imaged by a fully automated workflow in 10 rounds of staining utilizing one or two different fluorescent channels. Adapted with permission (Klevanski et al., 2020). **(C)** Exchange-PAINT imaging of four targets in primary neuron culture. Top shows the four-color overlay and zoom-ins with comparison to the diffraction-limited view. By determining the presynaptic and postsynaptic markers for inhibitory and excitatory synapses, the synaptic geometry can be visualized. Bottom shows a magnified view into single excitatory and inhibitory synapses, highlighting the side-by-side clustering of scaffold and marker proteins. Adapted with permission (Wang et al., 2017). **(D)** Exchange-PAINT imaging of four different targets in a calyx of Held tissue cryosection. Secondary Antibodies carrying four different docking handles were used for spectrally unlimited multiplexing. The middle panel shows the entire field of view with a zoom-in in the bottom panel and the overlayed image on the right side. Adapted with permission (Narayanasamy et al., 2021).

On the other hand, although the relative distribution of targets toward a reference can yield average protein target distributions, the characteristics of single cell diversity are lost. Ensemble data cannot evaluate true colocalization nor precisely dissect multiprotein clusters or complex structures. To allow super-resolved multiplexing in the same sample, most approaches use sequential staining and imaging techniques. Sequential staining allows for spectrally unlimited multiplexing by the removal of a probe signal post image acquisition and re-staining of the sample with the next target probe. Klevanski et al. (2020) implemented this approach in STORM and called it maS^3^TORM (Figure 2B). Using this strategy, the authors were able to multiplex 16 targets in the calyx of Held synapse (Klevanski et al., 2020). Although this sequential approach enables unlimited multiplexing, acquisition time increases substantially, as each antibody is incubated sequentially, which can take several hours or more per target. Also, the removal of the probe signal requires harsh treatments with SDS, generally followed by photobleaching reducing the sample quality with increasing multiplexing rounds.

DNA-PAINT offers an attractive, complementary, and relatively intuitive way to achieve spectrally unlimited super-resolution multiplexing by using different imager-docking handle pairs for imaging the individual targets. In this approach, which is called Exchange-PAINT (Jungmann et al., 2014), each protein target is labeled by an antibody with a unique docking sequence. For the multiplexing workflow, first only the transiently binding imager sequence to the first docking strand is flushed in and a super-resolution image is acquired. After this first round, the solution is washed out and the imager for the next target docking strand is incubated. The exchange of imager strands from one target to the next only takes a few minutes. In a first implementation of Exchange-PAINT to primary neurons, Wang et al. (2017) acquired super-resolved images of eight targets (Figure 2C). With colocalization analysis on the presynaptic active zone marker Bassoon, the inhibitory postsynaptic marker Gephyrin and the respective synaptic vesicle markers SynapsinI and VGAT, the authors were able to distinguish excitatory and inhibitory synapses and determine the geometric orientation of the synapses. The versatility of Exchange-PAINT has also been ported to STED and (d) STORM microscopy using slightly more stable imaging strands of about 12 nt, which label targets in a fixed manner during one image acquisition round. Originally demonstrated by Schueder et al. (2017) in HeLa cells, several groups have adopted the technique for diverse applications (Filius et al., 2021). A similar DNA exchange approach was also employed by Guo et al. (2019) The authors used DNA-labeled primary antibodies and DNA Exchange imaging to visualize the cross-sectional profiles of nine protein targets along the trans-synaptic axis (Figure 2A). In one recent study, the concept of Exchange-PAINT has also been applied to neuronal tissue sections. Using four-target Exchange-PAINT, Narayanasamy et al. (2021) were able to show the distribution of the pre- and postsynaptic scaffold proteins Bassoon and Homer1, as well as the glutamate vesicle marker VGlut1 in the calyx of Held synapse active zones with up to 25 nm resolution (Figure 2D).

While one of the major strengths of DNA-PAINT compared to other super-resolution techniques is the versatility in multiplexing by using programmable DNA barcodes, one substantial weakness of DNA-PAINT is its traditionally rather slow image acquisition process, practically limiting large-plex experiments. This limitation holds equally true for qPAINT, as precise counting is dependent on sufficient statistics to faithfully calculate averages from exponentially distributed times. Thus, generating sufficient statistics to truly approach the question of molecular organization in more than a few cells remains a challenge. To overcome this limitation, recent studies using secondary-structure-free sequences (Schueder et al., 2019) and sequence motif concatenation (Strauss and Jungmann, 2020) have improved DNA-PAINT’s image acquisition speed by a factor of 100. While the labeling of multiple targets using species-independent probes is technically possible for most biological samples, another factor might play a crucial role when investigating dense protein clusters: labeling issues with relatively large antibodies due to molecular crowding in dense clusters. If we roughly assume a size of approx. 15 nm for primary antibodies and use this to label synaptic vesicles with 50 nm size, the problem is not only the linkage error to the true target position, but also the potential blocking of binding sites for antibodies to other targets, making subsequent imaging rounds inevitably more challenging than the first one (or even impossible). To address this problem, the labeling probes and imaging rounds for multiplexed imaging need to be designed carefully, going from more sparse protein targets to more abundant and using smaller and better labeling probes.

## Discussion and Outlook

The major bottleneck for almost all quantitative and multiplexed SR applications to date is the specific and efficient labeling of the target proteins. We have discussed some of the more common approaches to address the labeling challenge (Moore and Legant, 2018). While they are quite versatile, both the labeling efficiency and stoichiometry are far from perfect. If researchers only aim to address a small number of targets, several different approaches can be implemented. Among the most sophisticated labeling approaches are the use of primary nanobodies, which combine all the advantages of stochiometric labeling and small linkage error, but they are only available for very few targets. Recent developments have introduced slow off-rate modified aptamers (SOMAmers) for DNA-PAINT applications. These small (7–30 kDa) synthetic DNA probes can be functionalized with a suitable docking sequence for single-molecule quantification (Strauss et al., 2018). Aptamers have furthermore been employed in DNA-PAINT imaging to characterize size and morphology of Amyloid-beta aggregates in human cerebrospinal fluid (De et al., 2019). If aptamers or primary nanobodies are not available, a hybrid approach featuring high labeling efficiency, low linkage error and stoichiometric labeling can be applied. Here, the protein of interest is genetically tagged, either with a fluorescent protein marker (Ries et al., 2012) or a small peptide tag (Virant et al., 2018; Gotzke et al., 2019), for which direct nanobody binders are available. These nanobodies have been evaluated in numerous studies and the versatility of genetic tagging enables the investigation of a broad range of protein targets. Further examples of genetically encoded probes are self-labeling enzymes such as SNAP-tag (Keppler et al., 2003) and HaloTag (Los et al., 2008), which can be combined with DNA-PAINT to enable 1:1 labeling of single proteins (Schlichthaerle et al., 2019). Furthermore, the use of monomeric streptavidin could help to further alleviate the labeling issue (Chamma et al., 2016). Of course, the trade off in the case of genetically encoded probes is the limited amount of multiplexing and the expertise and complexity it takes to generate a genetically tagged cell line or animal.

Unfortunately, at this point there is not a single most ideal solution for universal labeling. Practically every experiment and target requires a careful probe design and optimization workflow. There will inevitably be a trade-off between achievable resolution, accuracy of molecular position and quantification, and possible targets to multiplex. While remarkable progress has been achieved in the past years, major concerted efforts are required to develop small, efficient, and quantitative labels for future applications. Ever more powerful quantitative SR approaches in the future could lay the groundwork in investigating heterogeneous morphology, plasticity, and protein compositions in synapses as well as vesicle pools. An accurate mapping of receptor nanodomains and cytoskeleton structure related to the synaptic scaffold might provide a deeper understanding of signaling cascades. Lastly, the quantitative comparison of interaction patterns of key proteins in healthy and diseased tissue could lead to a more sophisticated understanding of major neurodegenerative and autoimmune diseases.

## Author Contributions

EU and RJ wrote the perspective. Both authors contributed to the article and approved the submitted version.

## Conflict of Interest

The authors declare that the research was conducted in the absence of any commercial or financial relationships that could be construed as a potential conflict of interest.

## Publisher’s Note

All claims expressed in this article are solely those of the authors and do not necessarily represent those of their affiliated organizations, or those of the publisher, the editors and the reviewers. Any product that may be evaluated in this article, or claim that may be made by its manufacturer, is not guaranteed or endorsed by the publisher.
